# How to Remove Scars

**Published:** 1885-03

**Authors:** 


### HOW TO REMOVE SCARS.

Scars on the face are always unsightly, and many occasion pain or inconvenience on account of their propensity to contract as they become older. The pressure on the nerves of the neighboring tissues by their constriction is sometimes an occasion of severe pain. Dr. Wark, of New York, asserts that scars may be removed or much altered by manipulation, which he directs to be made as follows: Place the ends of two or three fingers on a scar, if it be a small one, and on the margin if it be large, and vibrate the surface on the tissues beneath. The surface itself is not to be subjected to any friction; all the motion must be between the integument and the deeper parts. The location of the vibratile motion should be changed every ten or fifteen seconds until the whole scar has been treated, if it be of moderate size. If the scar be the result of a large scald or burn, the margins only should be treated at first; the advances towards the centre should be deferred until the nutrition of the margins has been decidedly improved. Only a little treatment should be applied to any one spot at the same time, but never with sufficient frequency or severity to cause pain. If the scar becomes irritable, suspend treatment until it subsides. In the course of two or three weeks, of faithful of treatment, the surface of the moderate size become more moveable, and will begin to form wrinkles like true skin when pressed from side to side. All these changes are due to improved nutrition, consequent on better blood circulation—the development of new sets of blood-vessels in the cicatricial tissue.

